# LAMP-2 absence interferes with plasma membrane repair and decreases *T*. *cruzi* host cell invasion

**DOI:** 10.1371/journal.pntd.0005657

**Published:** 2017-06-06

**Authors:** Natália Fernanda Couto, Dina Pedersane, Luisa Rezende, Patrícia P. Dias, Tayanne L. Corbani, Lívia C. Bentini, Anny C. S. Oliveira, Ludmila F. Kelles, Thiago Castro-Gomes, Luciana O. Andrade

**Affiliations:** 1 Department of Morphology/Federal University of Minas Gerais, Belo Horizonte, MG, Brazil; 2 Department of Biochemistry and Immunology/Federal University of Minas Gerais, Belo Horizonte, MG, Brazil; Instituto de Investigaciones Biotecnológicas, ARGENTINA

## Abstract

*Trypanosoma cruzi* enters host cells by subverting the mechanism of cell membrane repair. In this process, the parasite induces small injuries in the host cell membrane leading to calcium entry and lysosomal exocytosis, which are followed by compensatory endocytosis events that drive parasites into host cells. We have previously shown that absence of both LAMP-1 and 2, major components of lysosomal membranes, decreases invasion of *T*. *cruzi* into host cells, but the mechanism by which they interfere with parasite invasion has not been described. Here we investigated the role of these proteins in parasitophorous vacuole morphology, host cell lysosomal exocytosis, and membrane repair ability. First, we showed that cells lacking only LAMP-2 present the same invasion phenotype as LAMP1/2^-/-^ cells, indicating that LAMP-2 is an important player during *T*. *cruzi* invasion process. Second, neither vacuole morphology nor lysosomal exocytosis was altered in LAMP-2 lacking cells (LAMP2^-/-^ and LAMP1/2^-/-^ cells). We then investigated the ability of LAMP-2 deficient cells to perform compensatory endocytosis upon lysosomal secretion, the mechanism by which cells repair their membrane and *T*. *cruzi* ultimately enters cells. We observed that these cells perform less endocytosis upon injury when compared to WT cells. This was a consequence of impaired cholesterol traffic in cells lacking LAMP-2 and its influence in the distribution of caveolin-1 at the cell plasma membrane, which is crucial for plasma membrane repair. The results presented here show the major role of LAMP-2 in caveolin traffic and membrane repair and consequently in *T*. *cruzi* invasion.

## Author summary

*Trypanosoma cruzi* is the etiological agent of Chagas disease, a very debilitating illness that has no efficient treatment to date. Better knowledge of the mechanisms involved with host cell infection is important to change this scenario. *T*. *cruzi* enters host cells by subverting the mechanism by which cells repair small injuries in their plasma membrane. In this process, parasites interact with host cells causing membrane injuries. These injuries lead to secretion of lysosomal content to the extracellular media, which in turn causes the internalization of plasma membrane wounds via endocytosis. During this endocytic process the parasite is internalized by the host cell. We have previously shown that absence of two proteins located at the lysosomal membrane, LAMP-1 and 2, severely interferes with *T*. *cruzi* entry into host cells. In the present manuscript we show that absence of LAMP-2 alone is enough to compromise *T*. *cruzi* entry into host cells and it does so by compromising the endocytic events that follow lysosome secretion, which in turn is responsible for driving *T*. *cruzi* into the host cell interior.

## Introduction

*Trypanosoma cruzi* is the causative agent of Chagas disease. This parasite is naturally transmitted through the feces of an infected vector, a triatomine bug, but transmission may also occur through contaminated food, blood transfusion, placenta or organ transplantation [1]. Therefore, although originally endemic to Latin America, where the vector is widespread, Chagas disease is now found in non-endemic countries, especially in the southern part of the United States and Europe due to human migration [2–5]. Chagas disease is a serious and debilitating illness with a variable clinical course, ranging from asymptomatic to very serious cardiac and/or gastrointestinal disease [6]. Available treatment is not efficient, especially considering the chronic phase of the infection [7]. In order to survive and replicate in the vertebrate host, *T*. *cruzi* needs to interact with and invade host cells. Therefore the comprehension of the mechanisms involved in these processes is extremely important for the development of more efficient treatment and disease control.

The parasite is able to invade a wide variety of cell lines, professional and non-professional phagocytic cells. In order to gain entry into host cells, *T*. *cruzi* subverts the mechanism by which cells repair small injuries in their plasma membrane. These small membrane tears lead to extracellular calcium influx into the cell cytoplasm. Increase in intracellular calcium induces lysosome recruitment and fusion at the site of injury, which leads to the release of an enzyme called acid sphingomyelinase. This enzyme cleaves sphingomyelin, generating ceramide, which induces a compensatory endocytosis event that carries the damaged site to the cell interior, resealing the plasma membrane. In the case of *T*. *cruzi*, intracellular calcium increase may occur through a series of molecules released by the parasite or located at its surface, which will trigger host signaling events that promote a rise in intracellular calcium [8–12]. In parallel, *T*. *cruzi* is also able to induce microinjuries at the host cell membrane, leading to calcium influx from the extracellular media into the cells [13]. In both cases, calcium leads to lysosomal exocytosis at the site of parasite attachment/injury [14, 15], followed by compensatory endocytosis events that pull *T*. *cruzi* into host cells [13]. To the newly formed parasitophorous vacuole, more lysosomes fuse until the entire vacuole is covered with lysosomal membrane markers. Lysosomal association and fusion during *T*. *cruzi* host cell invasion is essential for the formation of a viable parasitophorous vacuole, without which parasites could exit host cells [15]. Lysosomes could be contributing to anchor and drag parasite into host cells. In fact, we have previously shown that cells lacking Lysosomal Associated Membrane Proteins 1 and 2 (LAMP1/2^-/-^ cells), the major integral membrane proteins of lysosomes, are less susceptible to *T*. *cruzi* infection [16]. However, the mechanism by which these proteins interfere with invasion is still unclear.

LAMPs are highly glycosylated proteins, rich in sialic acid, and estimated to cover approximately 80% of the lysosome luminal surface [17, 18]. Due to its high sialic acid content and the previously reported role of host cell sialic acid in *T*. *cruzi* invasion process [19–22], it has been suggested that LAMP’s sialic acid moieties could be contributing to the invasion phenotype in LAMP knock out cells [16]. However, despite being heavily sialylated and structurally very similar, LAMP-1 and 2 have only around 37% similarity in amino acid sequence [23, 24] and differ in function, as revealed by the generation of LAMP1 and 2 single knock out mice [18, 25–27]. Therefore we were interested in evaluating the real role of LAMP during *T*. *cruzi* invasion of host cells. In order to evaluate the mechanism by which LAMP proteins participate in *T*. *cruzi* host cell invasion, we decided to investigate the role of these proteins in known important events involved in this process: parasitophorous vacuole morphology, host cell lysosomal exocytosis, and membrane repair ability. For this, we used LAMP1/2^-/-^ cells in comparison to WT cells. In parallel, we performed the same assays using LAMP2 single knock out fibroblast (LAMP2^-/-^), since the lack of LAMP-2 caused a more severe alteration in cells and mice [28, 29].

## Materials and methods

### Antibodies and reagents

Anti–mouse LAMP-1 mAb (1D4B), as well as anti-mouse LAMP-2 mAb (ABL-93), were obtained from the Developmental Studies Hybridoma Bank. Anti-*T*. *cruzi* polyclonal antibodies were obtained from serum of rabbits immunized with *T*. *cruzi* trypomastigotes as described previously [30, 31]. Secondary antibodies, anti-rat IgG-Alexa fluor 488, anti-mouse-Alexa fluor 488 and anti-rabbit IgG-Alexa fluor 546 were obtained from Thermo Fischer Scientific.

### Cells and parasites

Mouse fibroblasts cell lines, derived from wild type (WT), LAMP1 and 2 (LAMP1/2^-/-^) or LAMP2 (LAMP2^-/-^) knock out C57BL6 mice, were obtained from a collection of cell lines from Dr. Paul Saftig’s laboratory (Biochemisches Institut / Christian-Albrechts-Universität Kiel, Germany), which were previously generated by spontaneous immortalization of primary fibroblasts in culture around passages 10–20 [18, 29, 32]. The cells were maintained in high-glucose DMEM (Thermo Fischer Scientific) supplemented with 10% fetal bovine serum (FBS), 1% penicillin/streptomycin (100U/mL and 100μg/mL, respectively) and 1% glutamine (DMEM 10%).

Tissue culture trypomastigotes (TCTs) from the *T*. *cruzi* Y strain were obtained from the supernatant of infected LLC-MK2 monolayers and purified as described by Andrews *et al*. (1987) [30].

### *T*. *cruzi* invasion assay

For invasion assays, 4x10^4^ cells (WT, LAMP1/2^-/-^ and LAMP2^-/-^ fibroblasts) in high glucose DMEM 10% were plated in each well of a 24-well plate, containing 13mm round glass coverslips. Cells were plated 24 h before the experiment and incubated at 37°C and 5% CO_2_. Cells were then exposed to *T*. *cruzi* TCTs Y strain for 20 min at 37°C at a multiplicity of infection (MOI) of 100. After parasite exposure, the monolayers were washed 4 times with phosphate buffered saline containing Ca^2+^ and Mg^2+^ (PBS+/+), in order to remove the non internalized parasites, and fixed in paraformaldehyde 4% overnight. After fixation, cells were processed for immunofluorescence.

### Immunofluorescence and quantification of parasite invasion

After fixation, coverslips with attached cells were washed three times in PBS, incubated for 20 min with PBS containing 2% BSA and processed for an inside/outside immunofluorescence invasion assay as described previously [30]. Briefly, extracellular parasites were immunostained with rabbit anti-*T*. *cruzi* polyclonal antibodies in a 1:500 dilution in PBS/BSA for 1h at room temperature, washed and labeled with Alexa Fluor-546 conjugated anti-rabbit IgG antibody (Thermo Fischer Scientific) in a proportion of 1:500 in PBS/BSA for 45min.

After the inside/outside immunofluorescence staining, host cell lysosomes were also immunostained using a 1:50 dilution of either rat anti-mouse LAMP-1 hybridoma supernatant (1D4B) or rat anti-mouse LAMP-2 hybridoma supernatant (Abl 93) in PBS/BSA/saponin and the appropriate fluorescent labeled secondary antibody anti-rat IgG-Alexa fluor 488, as described previously [15, 30]. After that, the DNA of host cells and parasites was stained for 1 min with DAPI (4',6-Diamidino-2-Phenylindole, Dihydrochloride—Sigma), 0,1μM in PBS, mounted, and examined on an Olympus BX51 microscope equipped with a Q color 3 camera controlled by the ImagePro Express Software (Olympus).

### Transmission Electron Microscopy and analyses of parasitophorous vacuole morphology

WT, LAMP1/2^-/-^ and LAMP2^-/-^ fibroblasts were infected with *T*. *cruzi* TCTs at a MOI of 100 for 20 min, washed in PBS+/+ and then fixed with 2.5% Glutaraldehyde in 0.1M PHEM buffer (5mM MgCl_2_.6H_2_O, 70mM KCl, EGTA 10mM, HEPES 20mM, PIPES 60mM), pH7.2. After fixation cells were gently scraped off with a rubber policeman, harvested by centrifugation and incubated in 3% low melting agarose. The hardened agarose with the sample was cut into small pieces and washed in PHEM buffer. All samples were post-fixed with 2% osmium tetroxide containing 1.5% potassium ferrocyanide in PHEM buffer for 1h at room temperature. The cells were dehydrated using increasing concentrations of graded acetone before embedding the pellet in Araldite. Ultrathin sections in 200 mash copper grids were stained with lead citrate and analyzed on Tecnai G2-12 –SpiritBiotwin FEI electron microscope. Quantification of vacuole volume density was performed by the ratio between the total area of the vacuole and the parasite area, using the Image J software. Values closer to 1 indicate a tight vacuole, while values greater than 1 indicate looser vacuoles.

### β-hexosaminidase assay

To evaluate the lysosomal exocytosis, a β-hexosaminidase secretion assay was performed according to previous work [31]. Briefly, WT, LAMP1/2^-/-^ and LAMP2^-/-^ cells were treated with Ionomycin (Calbiochem) 5 and 10 μM for 10 min at 37°C. After treatment, cell extracellular media was collected and cells were lysed with Triton x-100 (Sigma) 1% in PBS. Extracellular media and cell lysates were incubated with 50μL of β-hexosaminidase substrate, 6mM 4 methylumbelliferyl-N-acetyl-B-D-glucosaminide (Sigma-Aldrich), dissolved in Na-citrate-PO_2_ buffer (pH4.5). After 15–20 min of incubation at 37° the reactions were stopped by adding 100μL of stop solution (2M Na_2_CO_3_-H_2_O, 1.1M glycine). 100μL of this solution was used for reading at 365nm of excitation and 450 nm of emission in a spectrofluorimeter (Synergy 2, Biotek in the Center of Flow Cytometry and Fluorimetry, Department of Biochemistry and Immunology, ICB-UFMG).

### Compensatory endocytosis assay after cell injury

Compensatory endocytosis after injury was measured using a scrape wound assay followed by trypan blue fluorescence quenching, as previously described [33]. Briefly, WT cells were grown in 10cm plates, washed with HBSS at 4°C containing or not containing 1.8mM Ca^2+^ and labeled on the plasma membrane with 1μg/mL wheat germ agglutinin (WGA)–Alexa Fluor 488 (Invitrogen) for 1 minute at 4°C, followed by two more washes in HBSS. Cells were then wounded by scraping in the presence or absence of Ca^2+^ and incubated for 2 minutes at 37°C with 0.2% trypan blue before washing and FACS analysis. Due to WGA-Alexa Fluor 488 susceptibility to quenching by the membrane impermeable trypan blue, Alexa Fluor 488 fluorescence measurement will correspond to the amount of membrane endocytosed after trypan blue exposure.

### Acid sphingomyelinase exocytosis and detection

WT, LAMP1/2 and LAMP2 knockout cells were plated on 10 cm round dishes 24 hours prior to experiments at 2 x 10^6^ cells/dish. Cell monolayers were washed 3 X with PBS and incubated at 4^o^ C with 2 mL of cold HBSS media containing Ca^2+^. To induce PM damage, exocytosis of lysosomes and PM repair each cell type was scrapped at 4° C, gently re-suspended with a serological pipette and incubated at 37° C for 5 minutes to induce lysosomal secretion and plasma membrane repair. As controls, non-scraped cells were incubated for 5 minutes at 37° C and the media were collected to assess constitutive exocytosis. The suspensions containing the exocytosed content from lysosomes were transferred to ice and centrifuged for 30 minutes at 10,000 *g* in order to remove possible detached cells. The supernatants containing the enzymes released from lysosomes were concentrated 20 times using 3 kDa Amicon Ultra filter units and analyzed by SDS-PAGE and Western Blot. Immunoblot assay was performed using rabbit polyclonal anti-ASM (Abcam ab83354). Protein samples were prepared with reducing sample buffer, boiled, separated on 10% SDS-PAGE and blotted onto nitrocellulose membranes (Bio-Rad) using the Trans-Blot transfer system (Bio-Rad) for 1 hour and 30 minutes at 100 V. The membrane was blocked for 1 h with 5% milk solution. After incubation with the primary antibody and peroxidase conjugated secondary antibody, detection was performed using Luminata Forte Western HRP Substrate (Millipore) and a Fuji LAS-4000 Imaging System with Image Reader LAS-4000 software (Fuji).

### Membrane injury and repair assays

WT, LAMP1/2^-/-^ and LAMP2^-/-^ cells were cultured in 35mm culture dishes (3x10^5^ cells/dish) at 37^°^C in 5% CO_2_ in DMEM 10%. After 24 hours, cell monolayers, treated or not with MβCD, were washed at 4^°^C with Ca^2+^-free PBS followed by two more washes in Ca^2+^-free PBS. Cells were then incubated with HBSS containing Ca^2+^ at 4^°^C, wounded by scrapping and incubated for 5 minutes at 37^°^C, to allow plasma membrane repair. Afterwards, cells were incubated at 4^°^C for 5 minutes with Propidium Iodide (PI) (10μg/mL in HBSS). For hitting control, cells were incubated with Ca^2+^-free HBSS at 4^°^C, wounded by scraping in the presence of PI and incubated for 5 minutes at 37^°^C. Alternatively, cell monolayers were injured by incubation with DMEM 10% containing PI (10μg/mL) in the presence or absence of *T*. *cruzi* TCTs Y strain for 30 min at 37°C at a multiplicity of infection (MOI) of 100. After flow cytometry (FACS Scan; Becton Dickinson), the data were analyzed using FlowJo v10.1 software (Tree Star, Inc.).

### Actin cytoskeleton labeling

WT, LAMP1/2^-/-^ and LAMP2^-/-^ cells were plated on 24 well plates containing 13 mm round coverslips at a density of 5x10^4^ cells/well 24 hours before the assay. Cells were then fixed with paraformaldehyde (PFA) 4% for 1 hour at 4^°^C. After fixation, coverslips with attached cells were washed three times in PBS+/+, permeabilized with Triton X-100 0.1% (Sigma-Aldrich) and incubated for 30 min with PBS+/+ containing 1% BSA (PBS/BSA 1%). For labeling polymerized actin, cells were incubated with Phalloidin-Alexa Fluor 546 (Thermo Fischer Scientific) using a dilution 1:40 in PBS/BSA 1% at room temperature, followed by three additional washes. Labeled coverslips were mounted on glass slides and examined on a Zeiss Axio Imager.Z2 (ApoTome.2 structured illumination system) microscope.

### Plasma membrane cholesterol quantification

Plasma membranes (PM) from WT, LAMP1/2^-/-^ and LAMP2^-/-^ cells were separated from nuclei and large granule fraction through differential centrifugation and subsequently isolated from endoplasmatic reticulum using equilibrium density ultracentrifugation as previously described [34], with few modifications. After isolation, PM subfraction was saponified with 25 ml ethanolic sodium hydroxide (1M) under heating. The unsaponifiable phase (enriched in cholesterol) was extracted with ethyl acetate and dried out overnight. The unsaponifiable matter was treated with *N*,*O*-Bis(trimethylsilyl)trifluoroacetamide (BSTFA) to obtain trimethyl-silyl (TMS) derivatives [35]. Gas chromatography (GC) analyses were performed in a gas chromatograph HP7820A (Agilent) equipped with a flame ionization detector. An HP5 column (Agilent; 30 m x 0.32 mm x 0.25um) was used following a ramp protocol starting from 250°C at a rate of 10°C/min for 5 min, with an injector (split 1:30) at 300°C and a detector at 300°C. Hydrogen was used as the carrier gas (3 ml/min); and an injection volume of 3μl. Peak identification and cholesterol concentration were obtained comparing peak areas with a standard curve using known concentration of cholesterol (Sigma-Aldrich) derivatized under same conditions as samples. The final content of cholesterol was normalized by total protein concentration taken from particle free supernatant obtained during PM subfraction isolation and protein concentration was determined according to Lowry et al. using bovine serum albumin as standard [36].

### Caveolin detection

For Western blot detection of total caveolin-1 content, WT, LAMP1/2^-/-^ and LAMP2^-/-^ cells were plated the day before, scraped and lysed with RIPA buffer (25mM Tris-HCl pH 7.6, 150mM NaCl, 1% NP-40, 1% sodium deoxycholate, 0.1% SDS). Protein content was measured, samples were prepared with reducing sample buffer, boiled and 50 μg of protein were loaded in each lane on a 12% SDS-PAGE. After transfer (see above–same as for acid sphingomyelinase (ASM) detection) the membrane was incubated with anti-Caveolin 1 (BD–cat. # 610406) diluted 1:2000. Detection and imaging were performed as described for ASM detection.

For immunofluorescence labeling, WT, LAMP1/2^-/-^ and LAMP2^-/-^ cells were plated onto round glass coverslips the day before, fixed in 4% paraformaldehyde for 10 min and washed 3 x in PBS, followed by incubation for 45 min at room temperature in PBS containing 2% BSA and 0.05% saponin (PBS/BSA/saponin). Cells were then incubated for 2 h with anti-Caveolin 1 (BD–cat. # 610406) antibody diluted 1:500 in PBS/BSA/saponin, washed, followed by 1 h incubation with secondary anti-mouse antibodies conjugated with Alexa Fluor 488 (Thermo Fischer Scientific) diluted 1:500 in PBS/BSA/saponin. Coverslips were mounted using ProLong1Gold antifade reagent (Thermo Fischer Scientific) and imaged using a Zeiss Axio Imager.Z2 (ApoTome.2 structured illumination system) microscope with an Axiocam 503 monochrome camera controlled by the Zen Blue Software (Zeiss). Alternatively, cells were imaged in a Zeiss Axio Imager.Z2 Microscope to create a 3D reconstruction, which has been made following capture of 16 optical sections with an approximate 0.93μm interval, using the 63x oil objective. The 3D imaging stack has been reconstructed using Zen Blue Software.

## Results

### LAMP2^-/-^ cells are as refractory to *T*. *cruzi* invasion as LAMP1/2^-/-^ cells

We first decided to test the influence of lysosomal integral membrane protein 2, LAMP-2, in *T*. *cruzi* cell infection, when compared to LAMP1/2^*-/-*^ deficient or WT control fibroblasts. It had been shown before that LAMP-1 deficiency had little effect on cell morphology, metabolism or viability and led to overexpression of LAMP-2 in cells, suggesting that LAMP-2 might compensate for LAMP-1 deficiency [25]. On the other hand, LAMP-2 deficiency in mice led to a series of lysosomal defects, which together compromised mice viability [27, 28]. For this, we performed cell invasion assays using fibroblast cell lines generated from a LAMP2 knock out mouse (LAMP2^*-/-*^ fibroblasts) and compared to the invasion rates obtained from WT and LAMP1/2 double knock out mouse (LAMP1/2^*-/-*^ fibroblasts) [29]. The phenotypes of the cell lines were confirmed through immunolabeling with both anti LAMP-1 and LAMP-2 antibodies (S1 Fig).

**Fig 1:**
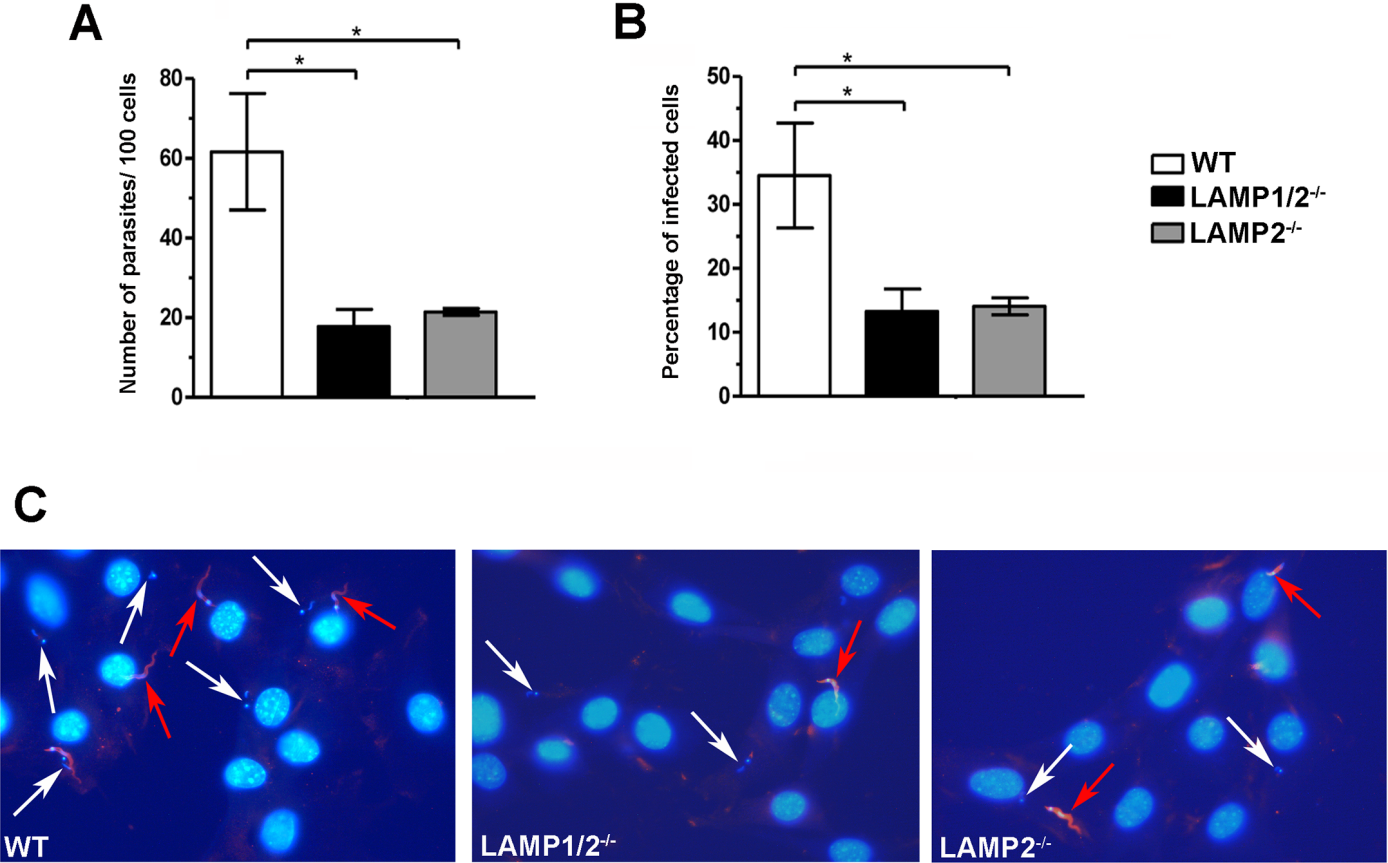
LAMP-2 deficiency is enough to compromise *T*. *cruzi* invasion in host cells. WT, LAMP1/2^-/-^ or LAMP2^-/-^ fibroblasts monolayers were exposed to Tissue Culture derived Trypomastigotes (TCT) from Y strain at a MOI of 50 for 20 minutes, washed, fixed and then processed for immunofluorescence detection of total intracellular parasites. Quantitative analysis of parasite infection rates in the three fibroblast cell lines was determined by the number of internalized parasites per 100 counted cells (A), as well as the percentage of infected cells (B). Data are shown as mean of triplicates ±SD. Asterisks indicate statistically significant differences (p<0.05, Student’s t test) between WT and LAMP deficient cells. (C) Representative panels of *T*. *cruzi* invasion in the three different fibroblast cell lines revealed by immunofluorescence labeling. Cell and parasite nuclei, as well as parasite kinetoplast DNA, were labeled with DAPI (blue); extracellular parasites in the field were labeled with anti-*T*. *cruzi* antibody followed by secondary IgG labeled with Alexa Fluor 546 (red). White arrows indicate intracellular parasites, while red arrows indicate extracellular parasites. Data shown are representative of three independent experiments.

As expected from previous data from our group [16], lack of both LAMP-1 and 2 led to a reduction in the invasion rate of *T*. *cruzi* TCTs when compared to WT fibroblasts (Fig 1A–1C). The number of internalized parasites per 100 counted cells (Fig 1A), as well as the percentage of infected cells (Fig 1B) in LAMP1/2^*-/-*^ fibroblast cultures are about 3 times lower when compared to their WT counterparts. The same was observed for LAMP2^-/-^ fibroblasts, revealing that absence of LAMP-2 alone was sufficient to reduce *T*. *cruzi* invasion rates to the same level observed for LAMP1/2^-/-^ fibroblasts and indicating a primary role for this protein in the invasion process (Fig 1A–1C).

### LAMP-2 deficiency does not affect *T*. *cruzi* parasitophorous vacuole morphology

**Fig 2:**
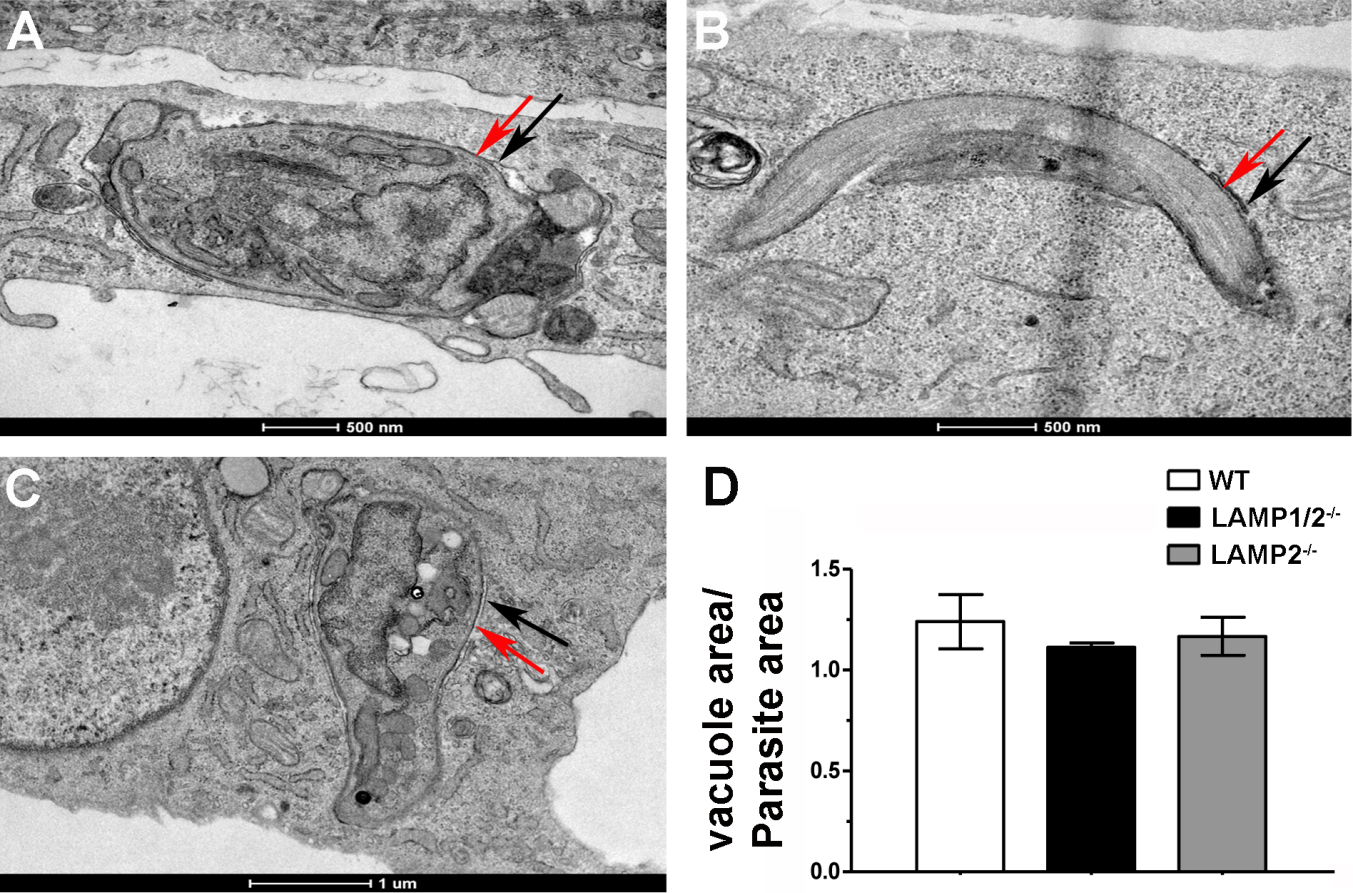
Absence of LAMP does not interfere with parasitophorous vacuole morphology. Transmission Electron Microscopy images of parasitophorous vacuoles from WT (A), LAMP1/2^-/-^ (B) e LAMP2^-/-^ (C) cells. Red arrows indicate parasite membrane and black arrows indicate parasitophorous vacuole membrane. (D) Graph shows the rate between parasitophorous vacuole and parasite areas. Data are shown as mean from 15 observed vacuoles ±SD from each cell line, WT, LAMP1/2^-/-^ and LAMP2^-/-^. No statistically significant differences were observed (P < 0,05, Student’s T test).

Interaction of *T*. *cruzi* with its vacuolar membrane has been shown to interfere with parasite invasion ability [22]. Therefore, we decided to test whether, in the absence of LAMP proteins, parasite interaction with its vacuole was compromised, contributing to the decreased cell invasion observed in LAMP knock out cells. For this, we evaluated parasitophorous vacuole morphology in the different cell lines (WT, LAMP1/2^-/-^ and LAMP2^-/-^ fibroblasts) through Transmission Electron Microscopy (TEM). TEM images of parasitophorous vacuoles containing recently internalized trypomastigotes, in the different cell lines (Fig 2A–2C), were processed using the software Image J and the ratio between the parasitophorous vacuole and parasite areas were calculated (Fig 2D). As expected, membranes of parasite and vacuole were tightly apposed to each other in WT cells, showing almost no intravacuolar space between parasite plasma membrane and parasitophorous vacuolar membrane (Fig 2A). The same was observed for LAMP1/2^-/-^ or LAMP2^-/-^ fibroblasts (Fig 2B and 2C, respectively). Quantification of the ratio between vacuolar area and parasite area confirmed that presence or absence of LAMP did not interfere with vacuolar morphology (Fig 2D). The ratio between parasitophorous vacuole and parasite areas for all three cell lines were very close to one, showing that the two membranes, parasite and vacuolar, were intimately associated even in the absence of LAMP.

### LAMP-2 deficiency does not impair lysosomal exocytosis

**Fig 3:**
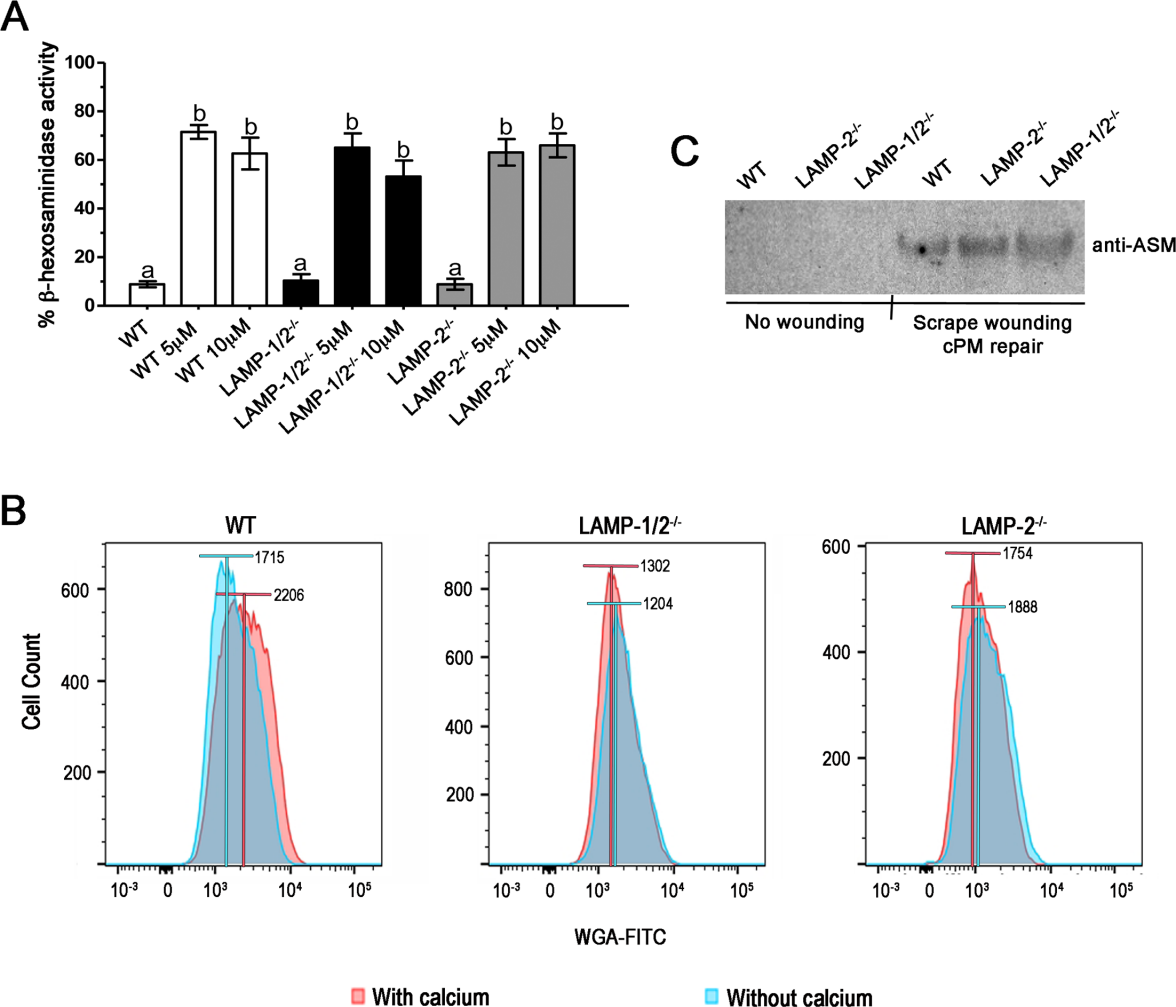
Absence of LAMP does not affect lysosomal exocytosis, but do interfere with compensatory endocytosis events. (A) Lysosome exocytosis assay. WT, LAMP1/2^-/-^ or LAMP2^-/-^ fibroblasts monolayers were exposed to 5 or 10μM Ionomycin for 10 minutes at 37°C, in the presence of calcium. Both extracellular media and lysates were collected and assayed for beta-hexosaminidase activity. Results are shown as the ratio between extracellular media β-hexosaminidase activity and total β-hexosaminidase activity (extracellular media plus cell lysate hexosaminidase activity). Non treated cells were used as lysosomal exocytosis negative control. Data are shown as mean of triplicates ±SD. Asterisks indicate statistically significant differences (p < 0.05, Student’s t test) between control and treated cells. (B) Measurement of compensatory endocytosis events induced by membrane injury. Cells were labeled with WGA-Alexa Fluor 488, submitted to membrane injury by cell scraping in the presence (red) or absence (blue) of calcium, and then incubated with trypan-blue to eliminate plasma membrane labeling. Only fluorescence from internalized membranes was preserved. The endocytosis was then quantified by FACS analysis. Histograms show the number of cells displaying WGA-Alexa Fluor labeling for the different fibroblast cell lines, WT, LAMP1/2^-/-^ and LAMP2^-/-^. Bars above the curves indicate the median of the fluorescence for each condition (with or without calcium). Data shown are representative of three independent experiments. (C) Detection of ASM in the supernatant of control non-scraped cells, and cells that had been subjected to scrape wounding and membrane repair. Supernatants of WT, LAMP1/2^-/-^ and LAMP2^-/-^, in the different conditions, were run on a gel, blotted onto nitrocellulose membranes and revealed using an anti-ASM antibody. The panel shows the presence of ASM in the supernatant of all three cells, only upon wounding and membrane repair.

As it is well known, *T*. *cruzi* invasion depends on lysosomal secretion induced by calcium signaling events [14, 37], followed by compensatory endocytosis, which drives parasites into host cells [13]. To test whether impairment in *T*. *cruzi* entry was due to deficiency in lysosomal exocytosis in cells lacking LAMP-2, we performed a lysosomal exocytosis assay using WT, LAMP1/2^-/-^ and LAMP2^-/-^ fibroblasts stimulated with Ionomycin, a calcium ionophore. Cells were exposed to Ionomycin in two different concentrations, 5 and 10μM and lysosomal exocytic events were measured by assaying β-hexosaminidase activity in cell culture supernatants. Supernatant of non-treated cells showed very little amounts of enzyme activity, as expected. On the other hand, treatment with Ionomycin did trigger lysosomal exocytosis in all cell lines, as it is demonstrated by the increase in β-hexosaminidase activity values in the cell supernatant upon treatment (Fig 3A). Treatment with 5 or 10μM of the drug led to the same exocytic values in all cell lines. Additionally and most important, no difference in lysosomal content release was observed among the distinct cell lines either before or after cell stimulation with the drug, indicating that absence of LAMP does not affect host cell lysosomal exocytic ability and could not contribute to the invasion phenotype observed for LAMP-2 deficient cells.

### LAMP-2 deficiency seriously affected compensatory endocytosis events induced by membrane injury

Once neither exocytosis nor vacuole morphology were compromised by LAMP deficiency, we decided to test whether compensatory endocytosis induced by these exocytic events, another important step of *T*. *cruzi* induced entry process [13], was affected. Compensatory endocytosis was measured by FACS analysis after scrape wounding using a trypan blue quantitative quenching assay [33, 38]. For this, WT, LAMP1/2^-/-^ and LAMP2^-/-^ fibroblasts were treated with WGA-Alexa Fluor 488, upon which cell membranes were fluorescently labeled, submitted to scrape wounding in the presence or absence of calcium and allowed to recover from injury. In this process, scrape wounding of plasma membrane will induce lysosome secretion, which will trigger compensatory endocytosis carrying the damaged WGA-Alexa Fluor 488 labeled membrane to cell interior. Cell external fluorescence was then quenched by trypan blue, to keep only fluorescence from internalized membranes, and cells were read using FACS. In the absence of calcium, endocytosis events were minimal, while in the presence of calcium endocytosis reached its maximum, enhancing intracellular WGA- Alexa Fluor 488 fluorescence. As observed in Fig 3B, a significant increase in intracellular WGA- Alexa Fluor 488 fluorescence is observed when WT cells were submitted to scrape wounding in the presence of calcium. However, no increase in intracellular WGA- Alexa Fluor 488 fluorescence was observed when LAMP1/2^-/-^ and LAMP2^-/-^ fibroblasts were submitted to the same procedure, indicating that LAMP-2 plays a critical role in the process of membrane repair by interfering with compensatory endocytosis after lysosome secretion (Fig 3B).

Since the endocytic process triggered by lysosomal secretion during membrane injury events is dependent on lysosomal acid sphingomyelinase (ASM) action on the extracellular leaflet of PM, we decided to evaluate whether ASM was present in the lysosomal secreted content during PM repair in WT, LAMP1/2^-/-^ and LAMP2^-/-^ cells. As shown in Fig 3C, the enzyme was normally exocytosed from lysosomes in all cell lines during resealing of mechanical wounds provoked by scraping. Thus, the defect in endocytosis observed in LAMP-2 deficient cells was not due to lack of ASM secretion and it is most likely due to events downstream of lysosomal secretion.

### LAMP absence interferes with membrane repair efficiency

**Fig 4:**
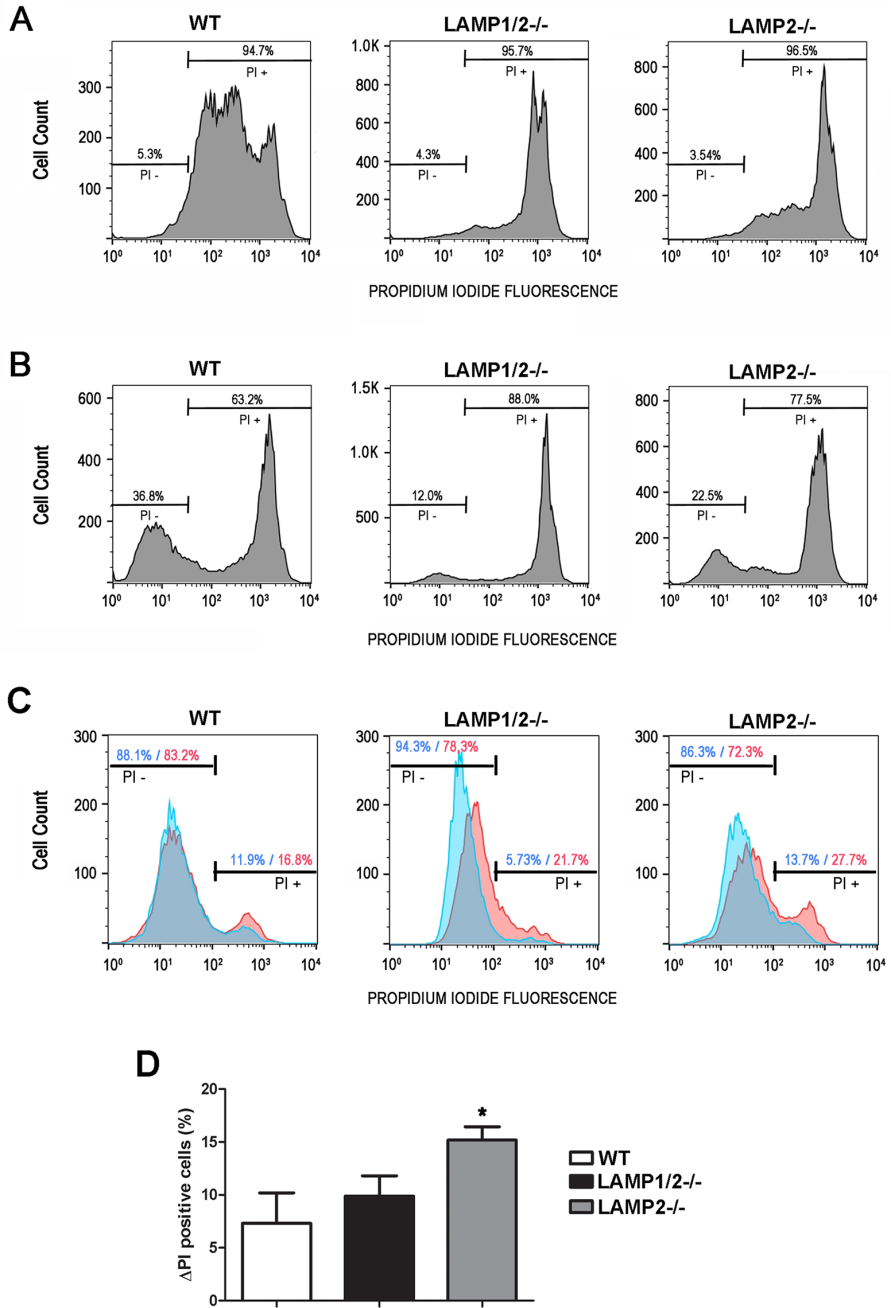
Absence of LAMP-2 does interfere with membrane repair. (A-B) WT, LAMP1/2^-/-^ or LAMP2^-/-^ were submitted to membrane injury by cell scraping. (A) Cells were scraped in the presence of Propidium Iodide (PI). Histograms show the number of cells presenting PI labeling (PI +), which represent the number of cells that suffered injury during scraping, while cells excluding PI represent those that didn’t suffer membrane injury. Bars above the curve indicate the percentage of injured (PI +) and non-injured cells (PI -). (B) Cells were scraped in the absence of PI, allowed to reseal, and then exposed to PI. Histograms show the number of PI + and PI–cells, which represent the ones that did not or did recover from injury, respectively. Bars above the curve indicate the percentage of non-viable (PI +) and viable cells (PI -). (C) Cells were exposed or not to *T*. *cruzi* trypomastigotes for 30 minutes in the presence of Propidium Iodide (PI). Blue curves represent control cultures, without parasite exposure, while red curves represent cell cultures exposed to *T*. *cruzi*. Histograms show the number of cells presenting PI labeling (PI +), which represent the number of cells that suffered injury, while cells excluding PI represent those that didn’t suffer membrane injury. Bars above the curve indicate the percentage of injured (PI +) and non-injured cells (PI -) in the presence or absence of *T*. *cruzi*. (D) Difference in the percentage of PI+ cells between control and *T*. *cruzi* exposed cell cultures. Asteriks indicate statistically significant differences p<0.05. Data shown are representative of three independent experiments.

If endocytosis induced during plasma membrane repair was compromised in LAMP-2 deficient cells we should also observe a defect in these cells' ability to repair their plasma membrane. To test whether cells lacking LAMP were really deficient in repairing injured membranes, an event that requires compensatory endocytosis, we performed a plasma membrane repair assay using the membrane impermeable fluorophore propidium iodide (PI). For this, cells were exposed to PI during or after scraping. In the first experiment, cells were exposed to PI during scraping to measure the efficiency in mechanical wounding of plasma membrane. Therefore, PI labeled cells provided the total number of cells injured by scraping from plates (Fig 4A). Scraping in the presence of PI resulted in 94.7% of WT cells injured by scraping, while only 5.3% were not injured in this process. A similar amount of injured/non-injured cells was found for LAMP1/2^-/-^ and LAMP2^-/-^ (95.7% / 4.3% and 96.5% / 3.5%, respectively). In the second experiment, cells were exposed to PI after scraping in order to measure cells' ability in repairing injured membrane. In this case, PI labeled cells provided the total number of cells unable to recover from membrane injury. On the other hand, cells that excluded the fluorophore included the cells that were never injured and the ones that had been injured, but were able to repair their membranes (Fig 4B). In order to calculate the percentage of cells that recovered from injury by membrane repair, for each cell type, we subtracted the percentage of not injured cells given by the first experiment (Fig 4A, PI-) from the total percentage of viable cells given by the second experiment (Fig 4B, PI-). As shown in Fig 4A and 4B, 5.3% of cells were negative for PI when exposed to the fluorophore during scraping, while 36.8% of cells were negative for PI when exposed to the fluorophore after scraping, indicating that 31.5% of cells recovered from injury by plasma membrane repair. On the other hand, only 7.7% (12% - 4.3%) of LAMP1/2^-/-^ cells and 19% (22.5% - 3.5%) of LAMP2^-/-^ cells were able to recover from membrane injury. This result confirms the importance of LAMP-2 for the membrane repair process.

In order to confirm that the decreased invasion in cells lacking LAMP-2 was due to their deficiency in compensatory endocytosis and not an inability of these cells to be injured by T. *cruzi*, we have also measured membrane injury, using the parasite as the source of membrane tear. For this, cells were exposed to *T*. *cruzi* trypomastigotes for 30 minutes in the presence of PI. Cultures not exposed to the parasite, but incubated with PI for the same amount of time, were used as controls in order to measure membrane injuries that may occur even in the absence of the parasite. As shown in Fig 4C, upon parasite exposure, it was possible to observe an increase in the number PI positive events for all cell lines (WT, LAMP1/2^-/-^ and LAMP2^-/-^) when compared to control condition (cell cultures without parasite exposure). WT cells exposed to *T*. *cruzi* showed about 7.3% more PI positive cells than its respective control, followed by LAMP1/2^-/-^ with 9.9% and LAMP2^-/-^ with 15.2% of PI positive cells (Fig 4D).

### LAMP absence interferes with cholesterol content, as well as caveolin-1, at the plasma membrane

**Fig 5:**
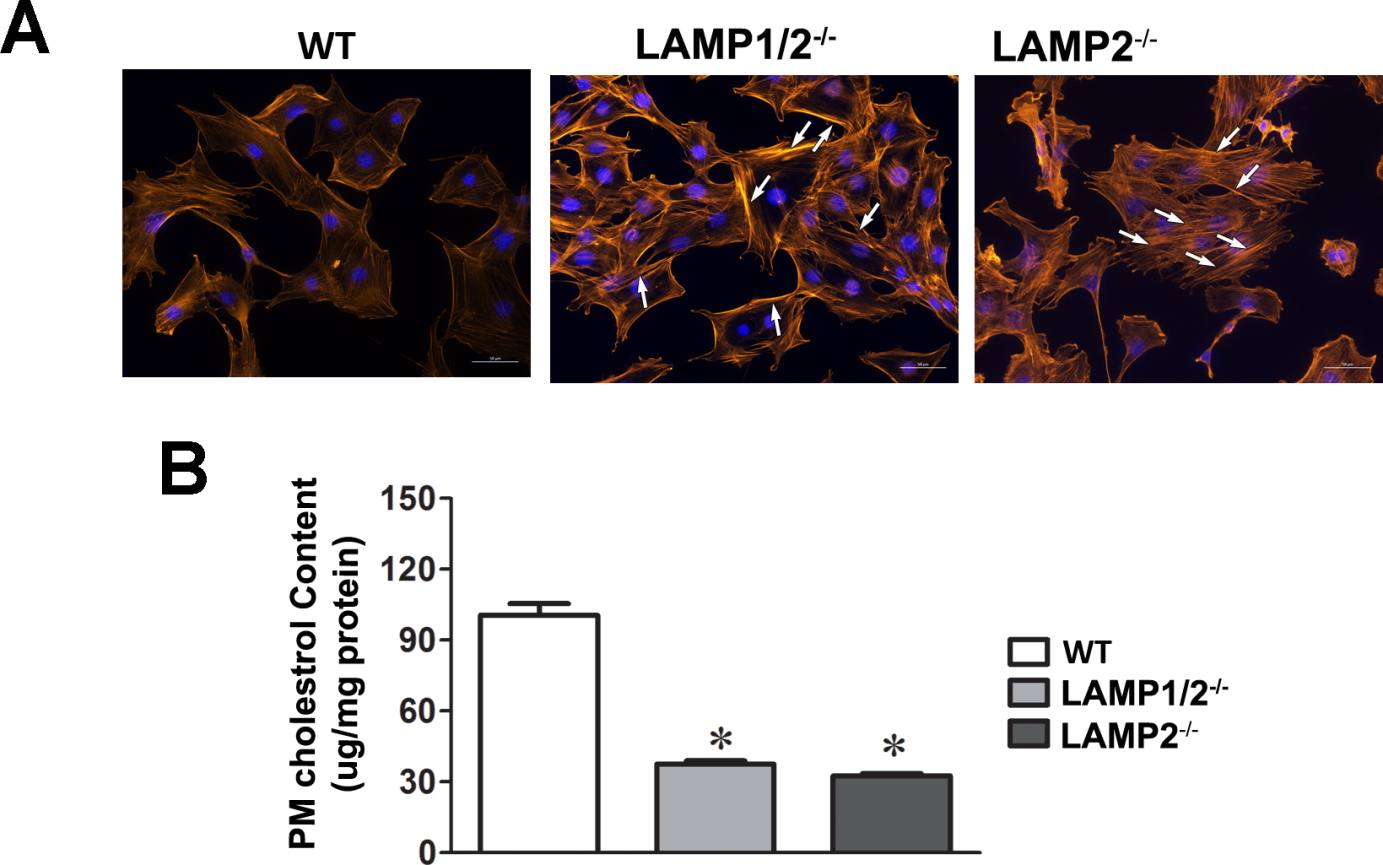
Absence of LAMP-2 leads to actin cytoskeleton rearrangement and decrease in cholesterol levels at the cell plasma membrane. (A) WT, LAMP1/2^-/-^ or LAMP2^-/-^ cells were submitted to phalloidin staining and analyzed in a fluorescence microscope. Arrows indicate the presence of the long actin stress fibers. (B) WT, LAMP1/2^-/-^ or LAMP2^-/-^ cells were submitted to lipid extraction from plasma membrane and the amount of cholesterol content evaluated. Plasma membrane cholesterol of LAMP2^-/-^ and LAMP1/2^-/-^ was measured as a percentage the plasma membrane content of WT cells, which was set as 100. Asterisk above bars indicate statistically significant differences.

LAMP absence, especially LAMP-2, had been previously shown to induce cholesterol accumulation in lysosomes [26, 29]. The latter could compromise the levels of cholesterol delivered to the cell plasma membrane and consequently interfere with caveolin-1 distribution at the cell surface, which is important for the compensatory endocytosis process triggered during membrane repair [39]. Since plasma membrane cholesterol sequestration using MβCD has been shown to induce actin stress fiber formation [31], we first decided to evaluate the actin cytoskeleton organization in WT, LAMP2^-/-^ and LAMP1/2^-/-^ using phalloidin staining of actin filaments. Analysis of the actin organization revealed that cells lacking LAMP-2 (LAMP2^-/-^ and LAMP1/2^-/-^) showed a differential organization of actin stress fibers in their cytoplasm, especially at the cell periphery, strongly suggesting that cholesterol content at the plasma membrane level was compromised in these cells (Fig 5A). In order to prove that the cholesterol content at the plasma membrane of cells lacking LAMP-2 was in fact lower when compared to WT cells, we prepared lipid extracts from cell plasma membrane and measured the cholesterol content. Cells lacking LAMP-2 showed a reduction of 70% in the levels of cholesterol at the cell plasma membrane when compared to WT cells (Fig 5B).

**Fig 6:**
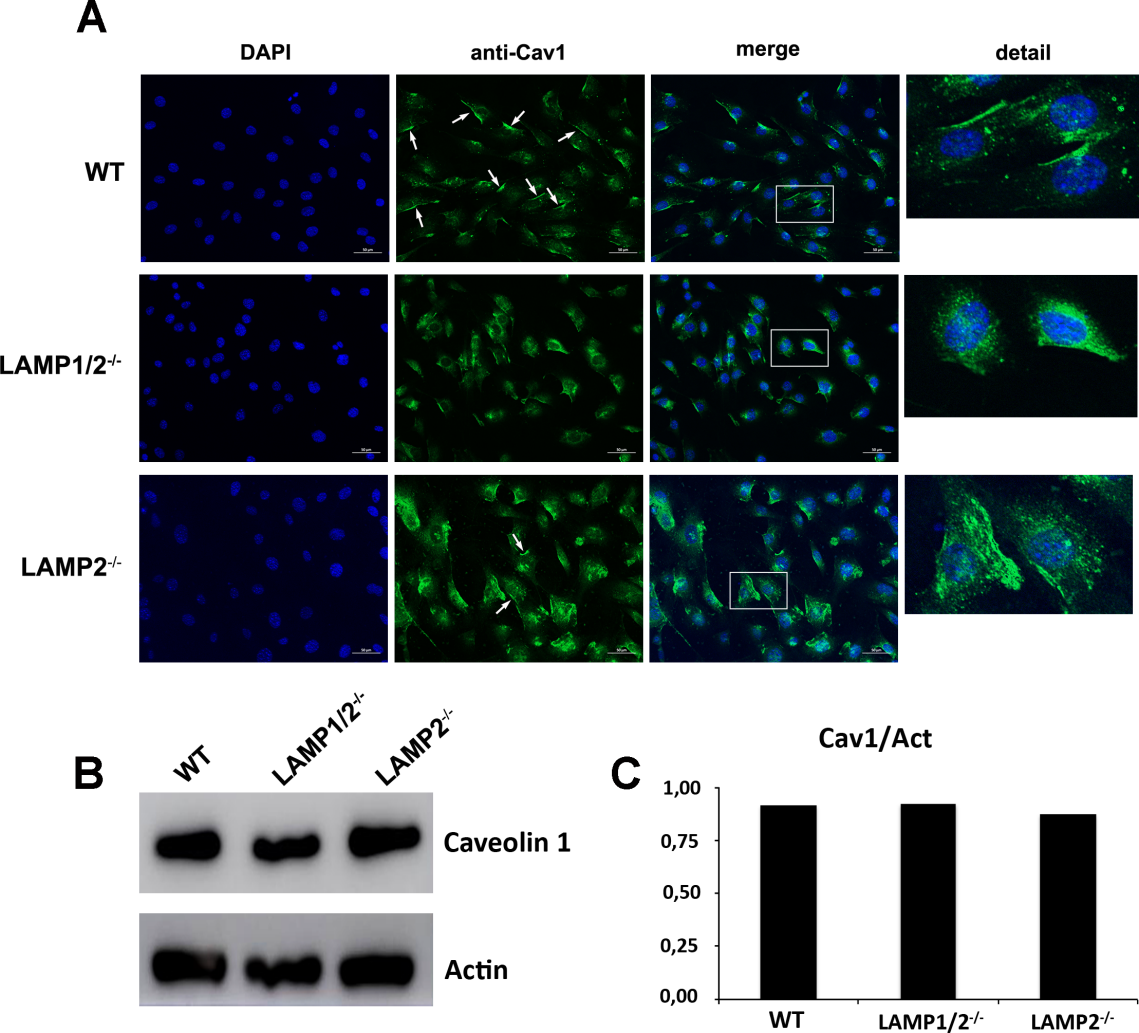
Absence of LAMP leads to decrease in caveolin associated with cell plasma membrane. (A) WT, LAMP1/2^-/-^, or LAMP2^-/-^ cells were fixed, submitted to labeling with anti-caveolin 1 and analyzed in a fluorescence microscope. Arrows indicate the presence of caveolin-1 at the cell surface. Details of each image are shown on the side. (B) WT, LAMP1/2^-/-^ or LAMP2^-/-^ cells were submitted to total protein extraction. Extracts were run on a gel, blotted onto nitrocellulose membranes and revealed using an anti-caveolin 1 antibody. The panel shows the presence of caveolin in the protein extracts from all three cells. (C) Graph shows the quantification of the amount of total caveolin in the different cell types.

We also labeled caveolin-1 using an anti-cav1 antibody and a secondary conjugated with Alexa-Fluor-488. WT cells show caveolin staining in cell interior, seen as small dots, as well as a very strong labeling at the cell surface (Fig 6A, S3 Fig and S1 Video). On the other hand, in cells lacking both LAMP-1 and 2 no surface labeling is observed, only the doted labeling in cell interior (Fig 6A, S3 Fig and S1 Video). LAMP2-/- cells show a profile similar to LAMP1/2^-/-^ cells, although in the first it is possible to see some labeling at the cell surface in few cells (Fig 6A, S3 Fig and S1 Video). In order to show that the amounts of caveolin produced by these cells were the same and only the distribution was different, we also evaluated by Western Blot the total amount of caveolin-1 in protein cell extracts. As it can be seen in Fig 6B and 6C, WT, LAMP1/2^-/-^ and LAMP2^-/-^ cells have the same amount of Caveolin-1.

## Discussion

In order to gain entry into host cells, *T*. *cruzi* stimulates them by interacting with their proteins and /or producing small injuries in their plasma membrane [10, 12, 40–42]. These events lead to the increase in intracellular calcium, which will in turn trigger lysosome exocytosis [43–45]. The latter is followed by a compensatory endocytic event that carries the parasite into the host cell [13]. Lysosomes have also been shown to be important for parasite retention inside cells [15]. Therefore these organelles have a pivotal role during *T*. *cruzi* invasion.

Lysosomal proteins LAMP-1 and 2 have been shown before to interfere with parasite invasion, since LAMP1/2 knock out cells led to very low levels of cell infection. LAMPs are not only the most abundant, but also highly glycosylated proteins, rich in sialic acid, and estimated to cover about 80% of the luminal surface of this organelle [17, 18, 46, 47]. Since sialic acid had been shown to be important for *T*. *cruzi* entry into host cells, it had been proposed that those residues could contribute to the observed LAMP knock out phenotype [16, 21, 48]. However, cells lacking only LAMP-2 were able to reproduce the invasion defect produced by abrogation of both LAMP proteins, strongly suggesting that LAMP-2 alone was responsible for the LAMP1/2^-/-^ invasion phenotype and that sialic acid moieties, specifically from LAMPs, were most likely not important for this process. This was reinforced by the fact that parasitophorous vacuole morphology was not altered in LAMP2^-/-^ or LAMP1/2^-/-^ cells as compared to WT cells. It had been shown before by Lopez and co-workers (2002) that cells lacking sialic acid are less susceptible to infection and that this phenotype was a result of the formation of a looser vacuole, where membranes of parasite and vacuole were not tightly apposed [22]. Altogether these data reinforced that intrinsic characteristics of LAMP-2, other than its sialic acid residues were important for *T*. *cruzi* invasion of host cells.

In order to investigate how LAMP-2 was involved with *T*. *cruzi* host cell invasion, we evaluated the different steps involved with parasite internalization. First we investigated the ability of these LAMP2 knock out cells to perform lysosomal exocytosis. It had been shown before that lysosome fusion with phagosomes was somewhat disturbed in LAMP1/2^-/-^ cells [49], indicating that lysosome mobility could be affected upon loss of LAMP. We showed that this was not the case, since upon stimuli these LAMP knock out cells were able to induce lysosome exocytic events. These data corroborate previous work from our group, which had shown that no parasite loss was observed in LAMP1/2^-/-^ cells [16], as would be expected when lysosomal fusion is blocked [15].

On the other hand we showed that compensatory endocytosis triggered by lysosomal exocytosis is compromised in cells lacking LAMP-2 (LAMP2^-/-^ and LAMP1/2^-/-^). These compensatory endocytic events are extremely important during membrane repair, since they are responsible for removing the injured membrane and promoting membrane resealing, without which cells would die [33, 38]. In fact, we showed here that LAMP-2 deficiency leads to more death of scraped injured cells. Although the latter could be explained by a compensatory endocytosis defect, it could also be a consequence of the fact that these cells are more prone to injury by scraping when compared to WT cells. The higher the number of injuries in one cell could lower its chance of membrane repair and recovery. In fact a larger number of cells presenting higher PI labeling values, was observed for cells lacking LAMP-2. The same was observed when we used the parasite as the source of injury. This susceptibility to membrane injury is probably linked to the fact that cells deficient in LAMP-2 retain cholesterol in lysosomes [29, 50]. LAMP-2 has been shown to bind cholesterol and help in its traffic to the plasma membrane [51], leading to less cholesterol at the cell surface, as demonstrated here. The decrease in the levels of cholesterol at the cell plasma membrane leads to actin cytoskeleton reorganization and cell stiffening [31, 52], which could make cells more prone to mechanical injury. We then tested whether cells pre-treated with MβCD, a drug able to decrease cholesterol from cell plasma membrane and induce actin cytoskeleton rearrangement would also lead to increased cell injury and death. In the conditions tested here, even though MβCD treated cells were more prone to injury by scraping, they were almost as efficient as non-treated cells in recovering from injury (see S2 Fig). Therefore, the inability of LAMP-2 deficient cells in recovering from injury should really be due to their inability to endocytose the injured membrane and not due to the fact that they are excessively injured.

We further investigated why compensatory endocytosis events were compromised in cells lacking LAMP-2. Compensatory endocytosis triggered by lysosomal exocytic events are dependent on the secretion of Acid Sphingomyelinase (ASM), an enzyme that cleaves sphingomyelin into ceramide inducing its coalescence and membrane internalization [33]. The levels and secretion of ASM were not altered in LAMP-2 deficient cells. This corroborates previous data by Eskelinen and coworkers (2004), which showed that although these LAMP-2 deficient fibroblasts present an accumulation of autophagic vacuoles no deficiency in protein degradation was observed, indicating that lysosomal enzyme content seemed to be unaffected by LAMP-1 or 2 deficiency [29]. Additionally, contrary to other cellular models of cholesterol storage defects, such as NPC (Niemann-Pick type C) patient cells, it has been demonstrated that LAMP1/2^-/-^ cells do not show significant differences in the levels of sphingomyelin, ceramide and gangliosides at the cell surface when compared to WT cells. Therefore, not only ASM but also its substrate were available to trigger endocytosis in cells lacking LAMP-2 [50]. Thus subsequent events had to be responsible for the compensatory endocytosis defect. LAMP1/2^-/-^ fibroblasts had also been shown to have a marked defect in the maturation of autophagosomes and phagosomes to degradative autolysosomes and phagolysosomes, indicating an alteration in lysosome mobility in these cells [29, 49, 53]. In the work by Huynh and coworkers, late endosomes/lysosomes, as well as phagosomes, show reduced ability to move on microtubules towards the cell center in LAMP1/2^-/-^ cells, most likely due to impairment in the interaction between them. This reduced mobility could also be accounting for the reduction in the observed endocytic events. However, it has also been shown that in LAMP1/2^-/-^ cells the phagosomes acquired Rab5 and accumulated phosphatidylinositol 3-phosphate normally, suggesting that the first steps of the endocytic pathways had not been altered when LAMPs are not present [49]. Moreover Schneede and co-workers have described that the maturation defect of autophagosomes and phagosomes to degradative autolysosomes and phagolysosomes in LAMP1/2^-/-^ cells was not observed in MEFs lacking only LAMP-1 or 2. Therefore mobility was not likely to be responsible for the reduced compensatory events observed for LAMP-2 deficient cells.

Corrote and coworkers described that the endocytosis of PM wounds is dependent on caveolin-1 and caveolar structures, this being a major mechanism used by cells to reseal plasma membrane injuries [54]. Interestingly, our results showed that the distribution of caveolin-1 at the cell surface is seriously compromised in cells lacking LAMP-2. Since caveolin-1 associates with cholesterol to form caveoale, cholesterol decrease in plasma membrane could be leading to dispersion of caveolin-1 as previously suggested [55]. Additionally, it has been shown that during caveolin traffic to the plasma membrane it accumulates in the medial Golgi, where it associates with cholesterol in order to be relocated to cell plasma membrane [56]. The altered intracellular traffic of cholesterol could be holding caveolin inside the cell and preventing its relocation to cell surface. In fact, we showed that the amount of caveolin produced in LAMP-2 deficient cells is the same as the one from WT cell. However, in LAMP-2 deficient cells caveolin is found only inside cells, apparently in small vesicles, most likely lysosomes as shown before [55]. Therefore, the lack of caveolin-1 at the plasma membrane could be responsible for the defect in compensatory endocytosis phenotype of LAMP2 knock out cells. This would also consequently account for less parasite internalization, especially considering that we have shown that *T*. *cruzi* was able to promote membrane injuries in all three cell lines, but failed to efficiently invade cells lacking LAMP-2.

The results shown here not only demonstrate the importance and mechanism by which LAMP-2 interferes with *T*. *cruzi* host cell entry but also indicate a major role for this protein in regulating plasma membrane repair. Consequently, it may help to understand the mechanisms involved with other diseases, related not only to LAMP-2 deficiency, such as Danon disease, but also with some lysosomal storage maladies or any genetic disorder in which impairment in plasma-membrane repair is observed.

## Supporting information

S1 Fig LAMP-1 and 2 labeling in WT, LAMP1/2^-/-^ and LAMP2^-/-^ cells, through immunofluorescence with anti-LAMP-1 (A, C and E) or anti-LAMP-2 (B, D and F) antibodies and secondary labeled with Alexa Fluor 488® (as described in Material and Methods, 2.4). Cell nuclei are labeled with DAPI. LAMP-1 and 2 labeling is observed in WT cells (A and B), while no labeling of LAMP-1 or 2 is seen in LAMP1/2^-/-^ fibroblasts (C and D). LAMP2^-/-^ fibroblasts show labeling for only LAMP-1 (E and F). Nuclei can be seen in all panels. Details of each panel are shown on the side.
(TIF)

S2 Fig Increased actin stress fibers make cells more susceptible to mechanical injury, but does not interfere with membrane repair. WT cells were treated with 5mM MβCD for 30 minutes in DMEM without serum and submitted to membrane injury by cell scraping. (A) Measurement of compensatory endocytosis events induced by membrane injury. Non-treated and MβCD-treated WT fibroblasts were labeled with WGA-Alexa Fluor 488, submitted to membrane injury by cell scraping, in the presence (red) or absence (blue) of extracellular calcium, and then incubated with trypan-blue to eliminate plasma membrane labeling. Only fluorescence from internalized membranes was preserved. The endocytosis was then quantified by FACS analysis. Histograms show the number of cells displaying WGA-Alexa Fluor 488 labeling. (B) Non-treated and MβCD-treated WT fibroblasts were either scraped in the absence of extracellular calcium and in the presence of Propidium Iodide (No Ca^2+^ + PI), to evaluate the amount of injury, or in the presence of extracellular calcium and absence of PI, allowed to reseal, and then exposed to PI (Ca^2+^ / PI), to evaluate the ability to recover from injury. For the “No Ca^2+^ + PI” condition, the number of PI+ cells represent the ones that suffered injury during scraping, while cells excluding PI represent those that didn’t suffer membrane injury. For the “Ca^2+^ / PI” condition, the number of PI- cells represent the ones did recover from injury and the PI+ cells the ones that did not recover from injury. Bars above the curve indicate the percentage of PI + and PI—cells. Data shown are representative of three independent experiments.
(TIF)

S3 Fig Z-stack images of WT, LAMP1/2^-/-^ and LAMP2^-/-^ labeled with anti-caveolin 1 antibody. WT, LAMP1/2^-/-^, or LAMP2^-/-^ cells were fixed, submitted to labeling with anti-caveolin 1 and imaged using a Zeiss Axio Imager Microscope. Eight optical slices with an approximate 1.86μm interval of each cell line were captured from bottom to top, using the 63x oil objective.
(TIF)

S1 Video 3D reconstruction of wild type, LAMP1/2^-/-^, and LAMP2^-/-^ cells. Cells were imaged in a Zeiss Axio Imager Microscope to create a 3D reconstruction, which has been made following capture of 16 optical slices with an approximate 0.93μm interval, using the 63x oil objective. The 3D imaging stack has been reconstructed using Zen Blue software.
(MP4)
