# Correction: LAMP-2 absence interferes with plasma membrane repair and decreases T. cruzi host cell invasion

**DOI:** 10.1371/journal.pntd.0008724

**Published:** 2020-09-01

**Authors:** Natália Fernanda do Couto, Dina Pedersane, Luisa Rezende, Patrícia P. Dias, Tayanne L. Corbani, Lívia C. Bentini, Anny C. S. Oliveira, Ludmila F. Kelles, Thiago Castro-Gomes, Luciana O. Andrade

The first author’s name is spelled incorrectly. The correct name is Natália Fernanda do Couto. The correct citation is: do Couto NF, Pedersane D, Rezende L, Dias PP, Corbani TL, Bentini LC, et al. (2017) LAMP-2 absence interferes with plasma membrane repair and decreases T. cruzi host cell invasion. PLoS Negl Trop Dis 11(6): e0005657. https://doi.org/10.1371/journal.pntd.0005657

The correct copyright statement is: © 2017 do Couto et al. This is an open access article distributed under the terms of the Creative Commons Attribution License, which permits unrestricted use, distribution, and reproduction in any medium, provided the original author and source are credited.
