# Supplementary figures and images for: LAMP-2 absence interferes with plasma membrane repair and decreases T. cruzi host cell invasion
Source: PLoS Negl Trop Dis. 2017 Jun 6;11(6):e0005657. doi: 10.1371/journal.pntd.0005657 (PMC5473579; doi:10.1371/journal.pntd.0005657)

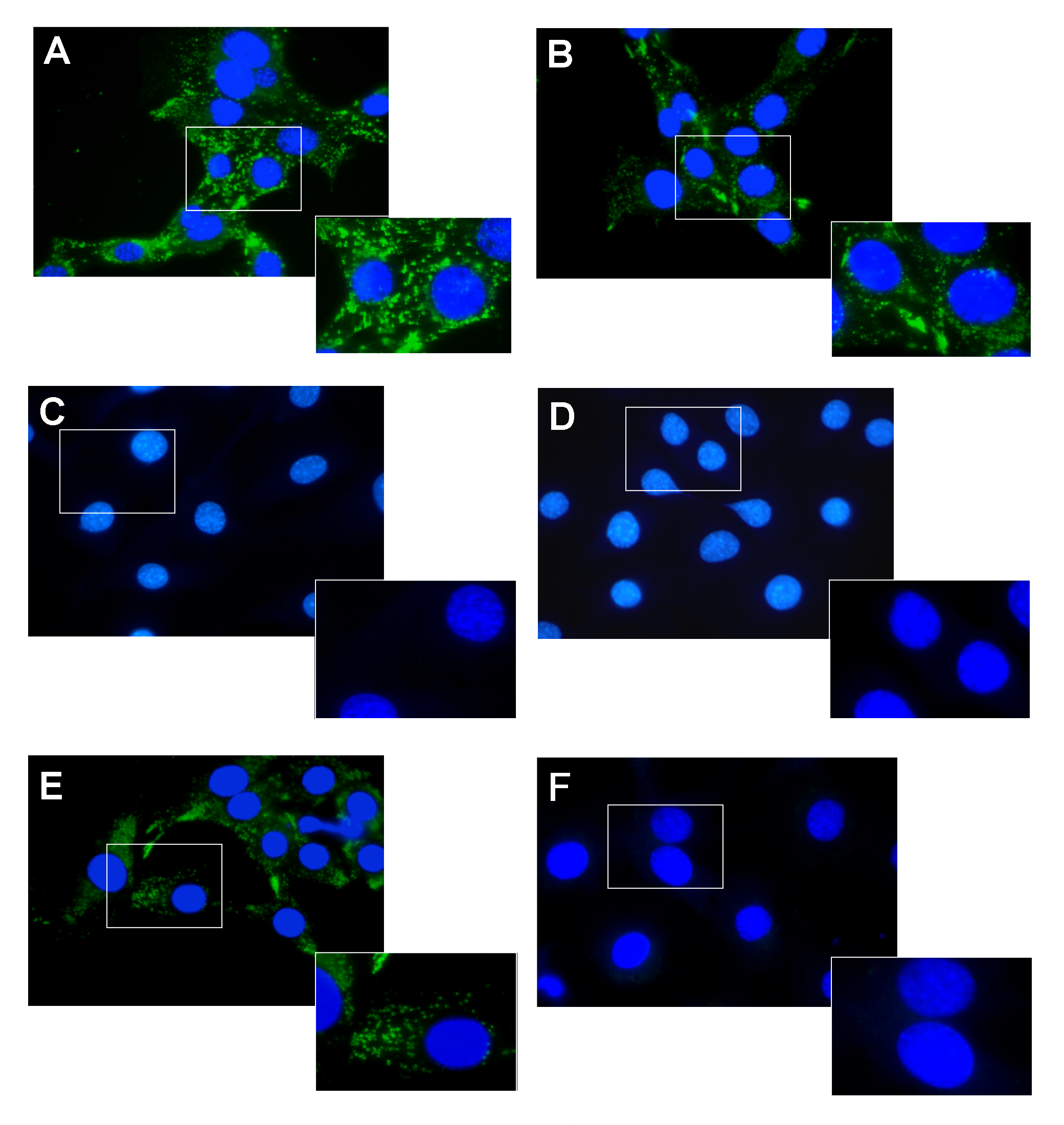

Supplement: S1 Fig — LAMP-1 and 2 labeling in WT, LAMP1/2-/- and LAMP2-/- cells, through immunofluorescence with anti-LAMP-1 (A, C and E) or anti-LAMP-2 (B, D and F) antibodies and secondary labeled with Alexa Fluor 488® (as described in Material and Methods, 2.4). Cell nuclei are labeled with DAPI. LAMP-1 and 2 labeling is observed in WT cells (A and B), while no labeling of LAMP-1 or 2 is seen in LAMP1/2-/- fibroblasts (C and D). LAMP2-/- fibroblasts show labeling for only LAMP-1 (E and F). Nuclei can be seen in all panels. Details of each panel are shown on the side. (TIF) [file pntd.0005657.s001.tif]

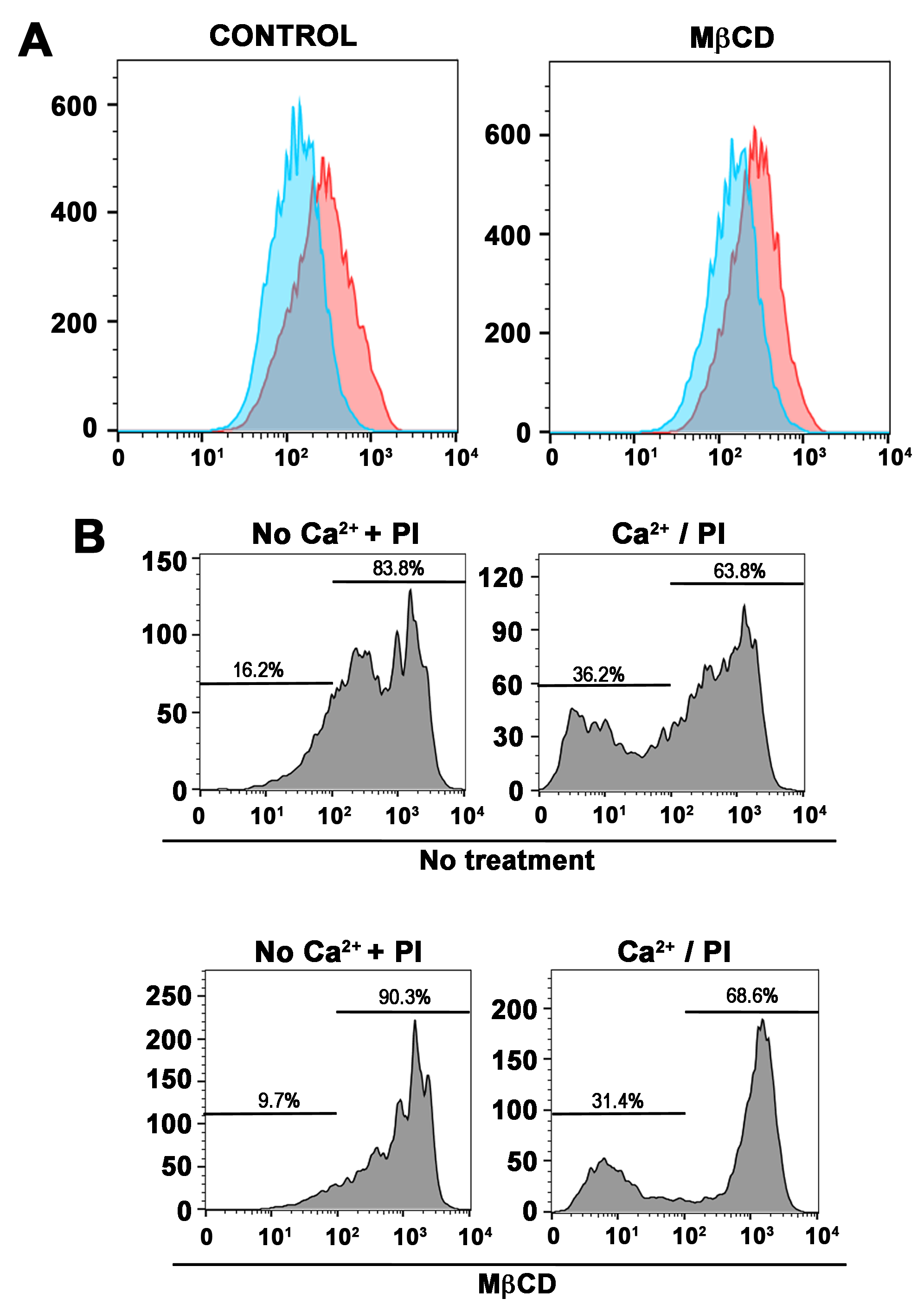

Supplement: S2 Fig — WT cells were treated with 5mM MβCD for 30 minutes in DMEM without serum and submitted to membrane injury by cell scraping. (A) Measurement of compensatory endocytosis events induced by membrane injury. Non-treated and MβCD-treated WT fibroblasts were labeled with WGA-Alexa Fluor 488, submitted to membrane injury by cell scraping, in the presence (red) or absence (blue) of extracellular calcium, and then incubated with trypan-blue to eliminate plasma membrane labeling. Only fluorescence from internalized membranes was preserved. The endocytosis was then quantified by FACS analysis. Histograms show the number of cells displaying WGA-Alexa Fluor 488 labeling. (B) Non-treated and MβCD-treated WT fibroblasts were either scraped in the absence of extracellular calcium and in the presence of Propidium Iodide (No Ca2+ + PI), to evaluate the amount of injury, or in the presence of extracellular calcium and absence of PI, allowed to reseal, and then exposed to PI (Ca2+ / PI), to evaluate the ability to recover from injury. For the “No Ca2+ + PI” condition, the number of PI+ cells represent the ones that suffered injury during scraping, while cells excluding PI represent those that didn’t suffer membrane injury. For the “Ca2+ / PI” condition, the number of PI- cells represent the ones did recover from injury and the PI+ cells the ones that did not recover from injury. Bars above the curve indicate the percentage of PI + and PI—cells. Data shown are representative of three independent experiments. (TIF) [file pntd.0005657.s002.tif]

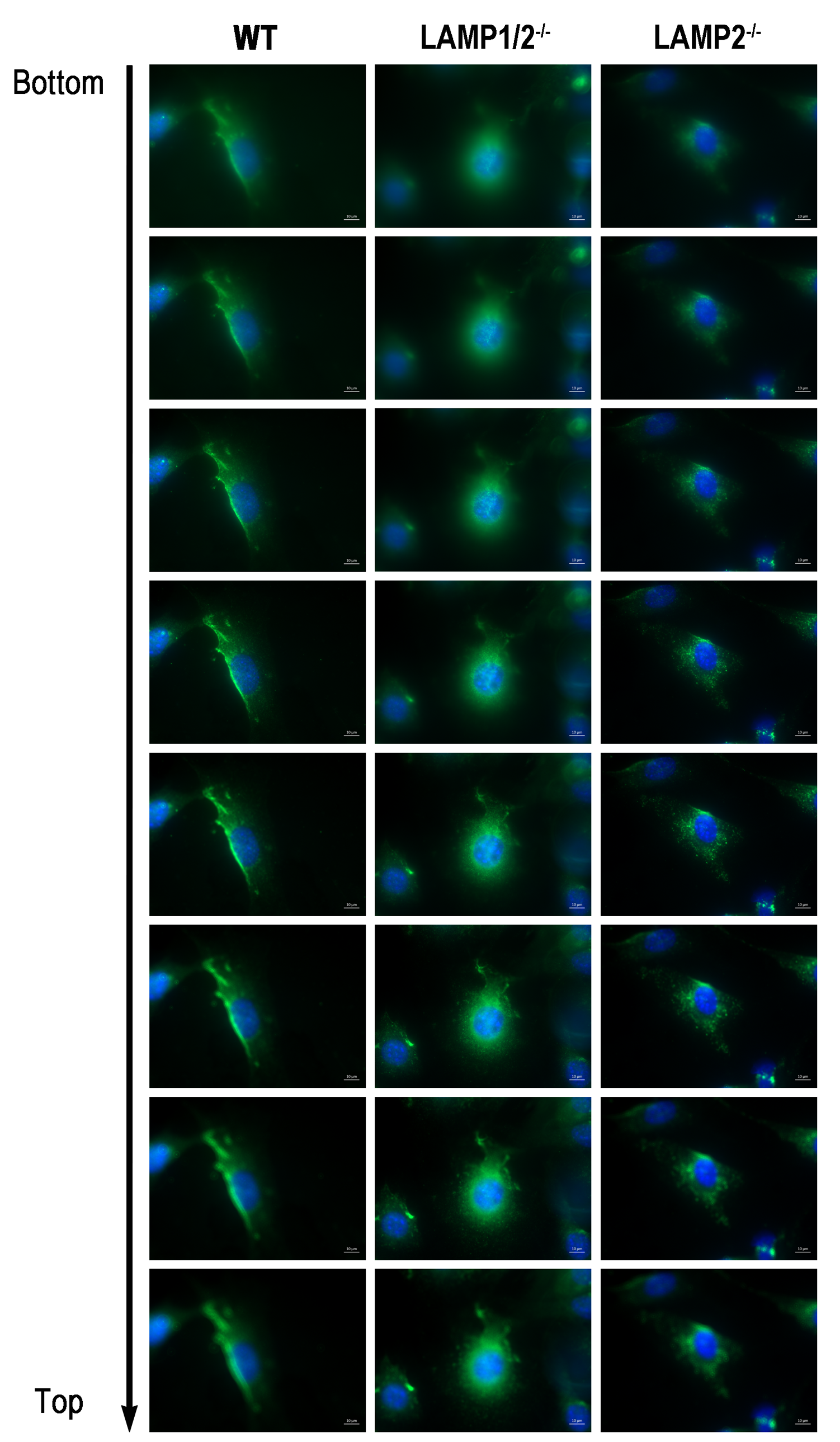

Supplement: S3 Fig — WT, LAMP1/2-/-, or LAMP2-/- cells were fixed, submitted to labeling with anti-caveolin 1 and imaged using a Zeiss Axio Imager Microscope. Eight optical slices with an approximate 1.86μm interval of each cell line were captured from bottom to top, using the 63x oil objective. (TIF) [file pntd.0005657.s003.tif]
